# Private sector antimalarial sales a decade after “test and treat”: A cross-sectional study of drug shop clients in rural Uganda

**DOI:** 10.3389/fpubh.2023.1140405

**Published:** 2023-03-28

**Authors:** Victoria Shelus, Nobert Mumbere, Edgar M. Mulogo, Clare Barrington, Emmanuel Baguma, Rabbison Muhindo, James E. Herrington, Michael Emch, Suzanne Maman, Ross M. Boyce

**Affiliations:** ^1^Department of Health Behavior, Gillings School of Global Public Health, University of North Carolina at Chapel Hill, Chapel Hill, NC, United States; ^2^Carolina Population Center, University of North Carolina at Chapel Hill, Chapel Hill, NC, United States; ^3^Department of Community Health, Faculty of Medicine, Mbarara University of Science and Technology, Mbarara, Uganda; ^4^Department of Epidemiology, Gillings School of Global Public Health, University of North Carolina at Chapel Hill, Chapel Hill, NC, United States; ^5^Department of Geography, University of North Carolina at Chapel Hill, Chapel Hill, NC, United States; ^6^Institute for Global Health and Infectious Diseases, University of North Carolina at Chapel Hill, Chapel Hill, NC, United States

**Keywords:** malaria case management, malaria diagnosis, drug shops, private health sector, rational drug use

## Abstract

**Background:**

The World Health Organization has promoted “test and treat” guidelines for malaria since 2010, recommending all suspected malaria cases be confirmed with a parasitological test, typically a rapid diagnostic test (RDT), prior to treatment with antimalarial medications. However, many fevers at private drug shops in Uganda continue to be treated presumptively as malaria without diagnostic testing.

**Methods:**

The purpose of this study was to document private sector malaria case management in rural Uganda through a cross-sectional survey of drug shop clients in Bugoye sub-county. Drug shop vendors (*n* = 46) recorded information about sales interactions with clients reporting fever or requesting antimalarials and collected capillary blood samples from clients who purchased medications without an RDT. We estimated the proportion of clients who purchased an RDT, adhered to the RDT result, and received antimalarials without having laboratory-confirmed malaria.

**Results:**

Most drug shops were unlicensed (96%) and sold RDTs (98%). Of 934 clients with suspected malaria who visited study drug shops during the data collection period, only 25% bought an RDT. Since some clients reported previous RDTs from the public sector, 40% of clients were aware of their malaria status at the drug shop. Among those with negative tests, 36% still purchased antimalarials. Sixty-five percent of clients who purchased an antimalarial without an RDT subsequently tested negative.

**Conclusions:**

Despite national guidelines, drug shop clients who purchase antimalarials from drug shops in Bugoye are often not tested to confirm a malaria diagnosis prior to treatment. Most clients treated presumptively with antimalarials did not have malaria. Interventions are needed to improve malaria case management and rational drug use in the private sector.

## 1. Introduction

Malaria remains a leading cause of death in low-income countries, despite considerable investment and effort toward elimination (1, 2). Globally, there were an estimated 241 million cases and 627,000 deaths attributed to malaria in 2020, with 96% of deaths occurring in sub-Saharan Africa (3). In Uganda, *Plasmodium falciparum* malaria is the leading cause of morbidity and mortality among all ages and is responsible for more than one quarter of inpatient deaths in children under 5 years of age (4). Malaria places a substantial burden on the Ugandan health system, accounting for 20–50% of pediatric outpatient visits (5), and has a negative socio-economic impact, with households spending up to 25% of their income on malaria prevention and treatment (4).

Early diagnosis and prompt treatment with antimalarial medications, specifically artemisinin-based combination therapy (ACT), are the primary strategies to prevent severe or fatal malaria complications (6–8). However, the non-specific symptoms of malaria, such as fever, headache, and myalgia, complicate clinical diagnoses (9). While malaria is often equated with a fever, other causes, particularly viral illnesses, account for most presentations (10–12). Therefore, the World Health Organization (WHO) has promoted “test and treat” guidelines since 2010, recommending that all suspected malaria cases be confirmed with a parasitological test, typically a rapid diagnostic test (RDT), prior to treatment (4, 6, 13). RDTs have proven effective for the timely and accurate diagnosis of malaria in many low-resource settings (14, 15).

Regardless of these policies, many fevers continue to be treated presumptively as malaria without confirmatory testing (16). Malaria overtreatment may occur if: (i) no diagnostic test is performed and fever is treated presumptively; (ii) the test result is negative but antimalarials are taken anyway; or (iii) the test result is falsely positive (17). Overtreatment wastes limited resources, delays treatment for other illnesses, and masks the true causes of disease within a population (9, 18, 19). Inappropriate antimalarial use also raises concerns about parasite resistance (9, 18, 19). *P. falciparum* has developed resistance to many previously used antimalarial medications (20), and artemisinin-resistant strains of *P. falciparum* have recently been reported from Uganda and Rwanda (21, 22).

In many low- and middle-income countries a substantial proportion of health services are provided outside the public healthcare system (23). In rural Uganda, where access to public health facilities is limited, private drug shops are commonly the first point of care for fever and an important source of antimalarials (24–27). Drug shops in Uganda can legally sell antimalarials and RDTs, but many shops are not licensed or regulated, and clients may purchase antimalarials without a confirmed malaria diagnosis (26, 28, 29). While RDT availability has increased recently, a 2015 national survey found less than one third of febrile drug shop clients received an RDT (30). Family or friends sometimes seek treatment on behalf of someone with suspected malaria, making it impossible to administer an RDT at the drug shop (30). Even when RDTs are used, clients do not always adhere to the results, purchasing antimalarials despite a negative test (26, 30).

The purpose of this study was to improve understanding of private sector malaria case management in Bugoye, western Uganda ~10 years after the Uganda Ministry of Health launched their “test, treat, and track” policy. To achieve this goal, we documented malaria diagnostic and treatment practices at drug shops, and then assessed the proportion of clients purchasing an RDT prior to the purchase of antimalarials, adhering to RDT results at the drug shop, and receiving antimalarials without having laboratory-confirmed malaria.

## 2. Materials and methods

### 2.1. Study setting

Bugoye is a malaria-endemic sub-county in rural western Uganda. The sub-county consists of 35 villages and has a population of ~42,000 (31). More than 80% of households rely on subsistence farming for their livelihood (32). The geography is characterized by deep river valleys and steep hillsides with elevations up to 2,000 meters. The tropical climate allows for year-round malaria transmission interspersed with semi-annual peaks after the rainy seasons (33). Malaria prevalence from the most recent Malaria Indicator Survey in the mid-western region of Uganda, which encompasses Bugoye, was estimated at 18% among children under 5 years of age (34), although more recent studies in the sub-county demonstrate substantial geographic heterogeneity (35).

Public health services in Bugoye are available from six level II health centers staffed by nurses and midwives, two level III health centers staffed by clinical officers, and community health workers (CHWs) who treat pneumonia, diarrhea, and malaria in children under 5 years of age. Level IV health facilities, staffed by physicians, are only available outside the sub-county. Given the remote nature of many villages in Bugoye, private drug shops play a large role in antimalarial distribution. Of nearly 4,000 encounters for antimalarials previously documented in 1 month, 53% sought care from drug shops, compared with 39% from health centers and 7% from CHWs (36). While several patient safety concerns emerged related to the type and dosage of antimalarials administered, no data was collected on the use of RDTs.

### 2.2. Study design

We conducted a cross-sectional survey of drug shop clients. Vendors recorded information about sales interactions with clients reporting fever or requesting antimalarials and collected capillary blood samples from clients who purchased medications without an RDT. Samples were subsequently transported to a laboratory and tested for malaria. Outcomes of interest included the proportion of clients who: purchased an RDT at the drug shop, purchased antimalarials after a negative RDT, and purchased an antimalarial without an RDT and subsequently tested negative for malaria.

### 2.3. Drug shop identification

A community sensitization meeting was held to discuss the objectives and methods of the study with local leaders and CHWs and enlist their help in identifying all the drug shops in their respective areas of the sub-county. The study team then visited the identified drug shops to provide information about the study and assess interest in participation. Drug shops were eligible to participate, regardless of licensing status, if they (i) were in Bugoye sub-county, (ii) sold any medications with an antimalarial effect, and (iii) vendors were willing to be trained on study procedures. Data on the professional background of vendors, years of operation, and the cost and type of RDTs sold was collected during initial drug shop visits (Supplementary material). Licensing status was verified with registration records at the Kasese District Health Office. Participating drug shops were divided into four groups based on geographic proximity, with all groups including a trading center and the surrounding villages. Each group completed training and data collection before the next group started training to reduce the logistical burden of collecting case report forms and blood samples over a relatively large geographic area.

### 2.4. Vendor training

Prior to implementation, participating vendors received a detailed study manual and completed a 90-min training on study procedures, including instruction on determining client eligibility, assigning ID numbers, completing data collection forms, obtaining informed consent, and collecting blood samples. A laboratory technician from Bugoye Health Center III demonstrated procedures for blood sample collection at each training. Finger-prick blood samples are widely employed to diagnose malaria within this setting, as they are required when using RDTs. Therefore, vendors who sell and administer RDTs were already familiar with this type of blood sample collection. Trainings were conducted by the study team in Ihukonzo, the local language. In accordance with national guidelines on COVID-19, all trainings were conducted outside with masks and social distancing. After the training, vendors collected data for 2 weeks. This data collection period was chosen based on budget and feasibility considerations, using previous antimalarial sales tracking in Bugoye to estimate client volume (36). At the end of data collection, vendors received a one-time stipend of 30,000 Ugandan Shillings (~$8.50) and a certificate for their participation.

### 2.5. Data collection and measures

The study was conducted from July to September 2021. All clients who visited participating drug shops during the data collection period reporting fever or purchasing antimalarials for themselves or another individual were eligible to participate. Drug shop vendors completed a paper data collection form for each eligible client. Data collection forms (Supplementary material) included information about the sales interaction (date, time), client demographics (village, age, sex, pregnancy status), brief clinical history (days of illness, symptoms), and medication purchases. Drug shop vendors recorded the results of RDTs purchased and performed at the drug shop, as well as any recent, client-reported results from RDTs conducted elsewhere (e.g., health center or CHW).

If the individual with fever (hereafter referred to as the “index client”) was present, did not purchase an RDT at the drug shop, and provided informed consent, vendors collected finger-prick blood samples (~0.2 mL) into Ethylenediaminetetraacetic acid (EDTA) microtainers (37). If a “surrogate client” was at the drug shop to purchase medications for someone sick at home, no blood sample was collected. Vendors were provided with all materials necessary to safely collect blood samples and dispose of waste. As a public health preventive measure, beginning with group two, clients who provided a blood sample were given a bar of soap to wash their hands. Blood samples were stored at the drug shop in insulated stainless-steel bottles packed with ice for up to 72 h.

### 2.6. Laboratory procedures

Blood samples from clients who did not purchase an RDT at the drug shop were tested for *P. falciparum* at Bugoye Health Center III by study staff using one of two RDTs: the CareStart Malaria Pf (HRP2) Ag RDT or the SD Biosensor Standard Q Malaria P.f. Ag test, based on local market availability. These RDTs detect the histidine-rich protein II antigen of *P. falciparum, are similar to kits used in routine clinical practice, and have* received pre-qualified status from WHO (38). RDTs were performed in accordance with the manufacturers' instructions and prior to the expiration.

### 2.7. Statistical analyses

Data was entered using REDCap electronic data capture tools hosted at the North Carolina Translational and Clinical Sciences Institute at the University of North Carolina at Chapel Hill (39). Data cleaning and statistical analyses were conducted in SPSS 28 (IBM Corp.) and SAS 9.4 (SAS Institute, Cary, NC). The primary outcome was the proportion of drug shop clients who purchased an antimalarial without an RDT result and subsequently tested negative for malaria. Secondary outcomes were the proportion of drug shop clients: seeking treatment for themselves vs. others, purchasing an RDT at the drug shop, knowing their malaria status (from RDTs conducted at the drug shop or elsewhere), adhering to the RDT results, and purchasing antimalarials or antibiotics. These outcomes were adapted from a systematic review of interventions introducing RDTs into private medicine retail outlets (40). We employed log-binomial regression modeling to estimate crude and adjusted risk ratios for having a known malaria status at the drug shop. Explanatory variables included client demographic factors, illness history, drug shop and vendor characteristics, RDT cost, visit date and time, and data collection group. Multivariate models included all explanatory variables.

## 3. Results

### 3.1. Drug shop characteristics

Forty-six eligible drug shops were identified in 20 villages, and all shops participated in the study. Half of the drug shops (*n* = 23, 50%) were concentrated in the three largest trading centers in Bugoye (Table 1). Drug shops had been operating for a median of 3 years (IQR: 1.8, 8.0), nearly all (*n* = 44, 96%) without a license from the Uganda National Drug Authority. Most drug shop vendors (*n* = 32, 70%) were trained as nursing assistants, which does not meet the necessary qualifications to operate a drug shop in Uganda. Nearly all drug shops sold RDTs (n=45, 98%), at a median price of 2,000 Ugandan Shillings (IQR: 2,000, 3,000) or ~$0.57 US Dollars. More than half of shops had RDTs in stock during the initial visit (*n* = 28, 61%). Eight types of RDTs were used with the most frequent being SD Bioline, SD Biosensor, Carestart, and First Response. All examined RDTs were valid based on the printed expiration date.

**Table 1:** Characteristics of drug shops that provide malaria treatment in Bugoye sub-county, Uganda.

	***N*** = **46**	
	***n***	**%**
	**med (IQR)**	
**Location** ^*^		
Small trading center	23	50.0
Large trading center	23	50.0
**Years of operation**		
<1 year	9	19.6
1–5 years	19	41.3
5–10 years	8	17.4
10+ years	10	21.7
**Licensing status** ^†^		
Unlicensed	44	95.6
Licensed	2	4.3
**Vendor training**		
Nursing assistant	32	69.6
Nurse or midwife	14	30.5
**Availability of RDTs**		
Yes	45	97.8
No	1	2.2
**Malaria RDTs in-stock** ^‡^		
Yes	28	60.9
No	18	39.1
**Malaria RDT cost**	2,000 UGX^§^	
	(IQR: 2,000, 3,000)	

* Large trading centers were defined by the presence of a weekly market day.

† License to operate a drug shop issued by the National Drug Authority.

‡ On the day of initial drug shop visits in May, June, or July 2021 (prior to data collection).

§ Approximately 0.57 USD.

### 3.2. Drug shop clients

During the data collection period, 934 clients visited drug shops in Bugoye reporting fever or requesting antimalarials. Many clients (*n* = 410, 44%) came to the drug shop outside normal business hours (i.e., 9 a.m. to 5 p.m.), including 28% in the evening and 24% over the weekend. Drug shop clients were evenly split by sex and had a median age of 21 years (IQR: 12, 32) (Table 2). Few clients were among the highest risk groups for severe malaria outcomes—only 11% of clients were under the age of five, while 4% reported being pregnant. The median length of time clients had been sick prior to coming to the drug shop was 3 days (IQR: 2, 4), most commonly with fever (*n* = 832, 89%), headache (*n* = 747, 80%), and joint or muscle pain (*n* = 560, 60%).

**Table 2:** Characteristics of clients seeking malaria treatment from drug shops in Bugoye sub-county, Uganda^*^.

	**All clients**, ***N*** = **934**		**Index client present at drug shop**, ***N*** = **662**		**Surrogate client present at drug shop**, ***N*** = **272**	
	***n***	**%**	***n***	**%**	***n***	**%**
**Sex of client**						
Male	448	48.0	305	46.1	143	52.6
Female	485	52.0	356	53.9	129	47.4
Missing	1		1			
**Age of client**						
Under 5 years	96	10.5	73	11.2	23	8.6
5–14 years	194	21.2	104	16.0	90	33.8
15–49 years	566	61.7	428	65.7	138	51.9
50–69 years	50	5.5	35	5.4	15	5.6
70+ years	11	1.2	11	1.7	0	0.0
Missing	17		11		6	
**Individual at drug shop**						
Person who is sick	662	70.9	662	100.0	0	0.0
Family member or friend	272	29.1	0	0.0	272	100.0
**Days of illness**						
0–1 days	129	14.5	83	13.1	46	17.9
2–3 days	478	53.6	332	52.3	146	56.8
4–5 days	179	20.1	139	21.9	40	15.6
6–7 days	79	8.9	60	9.4	19	7.4
More than 1 week	27	3.0	21	3.3	6	2.3
Missing	42		27		15	
**Presenting symptoms**						
Fever	832	89.1	582	87.9	250	91.9
Headache	747	80.0	536	81.0	211	77.6
Joint or muscle pain	560	60.0	413	62.4	147	54.0
Cough	357	38.2	252	38.1	105	38.6
Nausea or vomiting	204	21.8	158	23.9	46	16.9
Shivering/chills	132	14.1	102	15.4	30	11.0
Fatigue	113	12.1	88	13.3	25	9.2
Diarrhea	97	10.4	68	10.3	29	10.7
**Day of drug shop visit**						
Weekday	695	75.6	503	77.3	192	71.6
Weekend	224	24.4	148	22.7	76	28.4
Missing	15		11		4	
**Time of drug shop visit**						
Morning	314	34.7	230	35.9	84	31.7
Afternoon	342	37.7	244	38.1	98	37.0
Evening	250	27.6	167	26.1	83	31.3
Missing	28		21		7	

* Data collected between July 12 and September 22, 2021 from 46 drug shops in Bugoye sub-county. Drug shop clients were eligible to participate in the study if they presented during the data collection period reporting fever or requesting antimalarial medications.

In most cases, the index client came to the drug shop to purchase their own medications (*n* = 662, 71%), while 29% of clients purchased medications for others. If a surrogate client was at the drug shop (*n* = 272), it was often a parent seeking treatment for their child (*n* = 125, 46%). Surrogate clients also included siblings (*n* = 56, 21%), spouses (*n* = 38, 14%), children (*n* = 34, 13%) and friends (*n* = 8, 3%). Index clients present at the drug shop were significantly older (24.5 ± 15.8) than those who sent a surrogate (21.1 ± 14.9), *t*_(915)_ = −3.067, *p* = 0.002. There were no significant differences in presence of the index client at the drug shop by sex, days of illness, or visit day and time.

**Figure 1:**
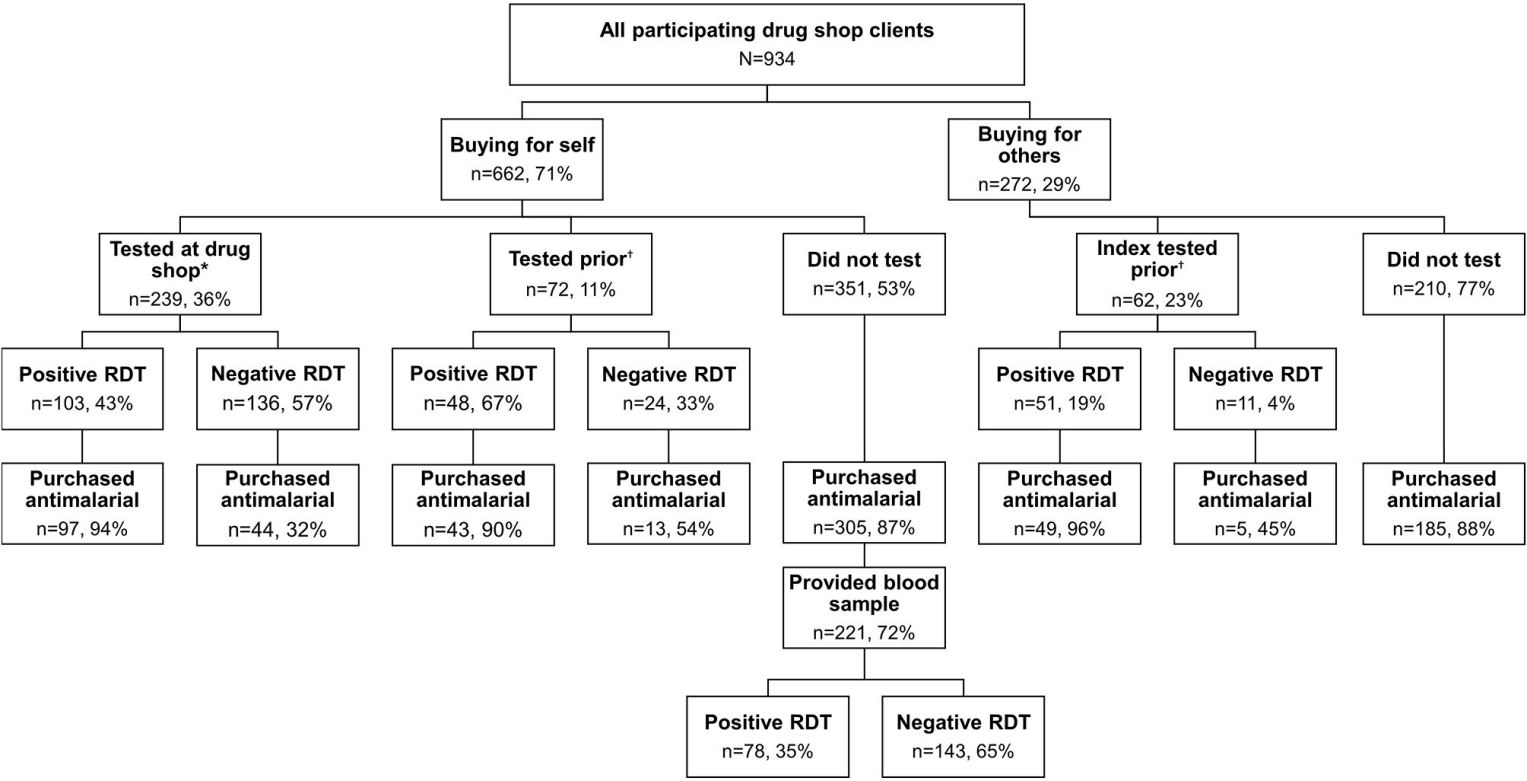
Use of malaria rapid diagnostic tests among clients seeking malaria treatment from drug shops in Bugoye.

Approximately one quarter of all clients (*n* = 239, 26%), and 36% of index clients present at the drug shop, purchased an RDT at the drug shop (Figure 1). Additionally, 20% of clients reported prior RDT results from a health center IV (*n* = 4, 2%), health center III (*n* = 49, 29%), health center II (*n* = 75, 44%), CHW (*n* = 21, 12%), or another drug shop or private clinic (*n* = 20, 12%). In total, 40% of clients were aware of their malaria status while purchasing medications. Of those with a test result, 54% tested positive for malaria (*n* = 202), while 46% tested negative (*n* = 171).

**Figure 2:**
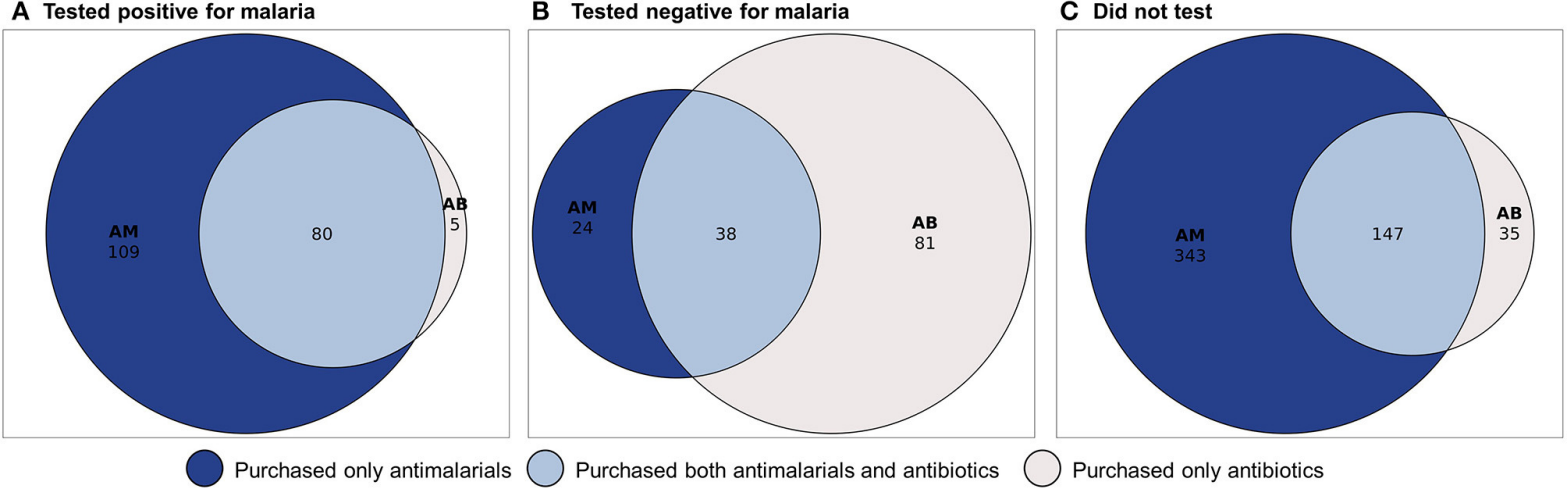
**(A–C)** Antimalarial and antibiotic purchases of drug shop clients by malaria test status.

Most clients purchased an antimalarial (*n* = 741, 80%), specifically artemether/lumefantrine (*n* = 499, 53%), sulfadoxine/pyrimethamine (*n* = 118, 13%), quinine (*n* = 76, 8%), artesunate (*n* = 34, 4%), dihydroartemisinin/piperaquine (*n* = 19, 2%), and chloroquine (*n* = 1, <1%). The majority were oral antimalarials, though 6% of clients (*n* = 60) purchased intravenous antimalarials (artesunate or IV quinine). Additionally, 41% of clients purchased antibiotics, commonly amoxicillin (*n* = 240, 26%), ampicillin (*n* = 58, 6%), and erythromycin (*n* = 51, 6%). Twenty-eight percent of clients purchased both antimalarials and antibiotics at the drug shop (Figure 2).

Among clients who knew they were positive for malaria at the drug shop, most purchased antimalarials (*n* = 189, 94%) and/or analgesic/antipyretic medications (*n* = 167, 83%), though 40% purchased both antimalarials and antibiotics (Table 3, Supplementary material). Clients who tested negative at the drug shop or another location commonly purchased analgesic/antipyretic medications (*n* = 158, 92%) or antibiotics (*n* = 119, 70%), but more than one third purchased antimalarials despite their negative result (*n* = 62, 36%), and 22% purchased both antimalarials and antibiotics (*n* = 38). Most clients with an unknown malaria status at the drug shop purchased antimalarials (*n* = 490, 87%) and/or analgesic/antipyretic medications (*n* =5 05, 90%). Approximately one third of clients who did not test for malaria at the drug shop purchased antibiotics (*n* = 182, 32%), and one quarter purchased both antibiotics and antimalarials (*n* = 147, 26%).

**Table 3:** Medication purchases of clients seeking malaria treatment from drug shops in Bugoye sub-county, Uganda by known test status at the drug shop^*^.

	**Tested positive for malaria**, ***N*** = **202**		**Tested negative for malaria**, ***N*** = **171**		**Did not test**, ***N*** = **561**	
	***n***	**%**	***n***	**%**	***n***	**%**
**Type of medication purchased**						
Antimalarial^†^	189	93.6	62	36.3	490	87.3
Antibioti^‡^	85	42.1	119	69.6	182	32.4
Analgesic/Antipyretic^§^	167	82.7	158	92.4	505	90.0
None	2	1.0	10	5.8	7	1.2
Purchased both antimalarial and antibiotic medications	80	39.6	38	22.2	147	26.2

* Includes both RDTs conducted prior to drug shop visit and RDTs conducted at the drug shop.

† Antimalarials included artemether/lumefantrine (Coartem, Lonart), sulfadoxine/pyrimethamine (Fansidar), quinine (in any form—tablet, IV, or syrup), artesunate, dihydroartemisinin/piperaquine (P-Alaxin, Duo-Cotecxin), and chloroquine.

‡ Antibiotics included: amoxicillin, ampicillin, erythromycin, metronidazole, co-trimoxazole, ciprofloxacin, ceftriaxone, gentamicin, benzylpenicillin, penicillin V, amplicox, cefalexin, tinidazole, azithromycin, and chloramphenical.

§ Analgesic/Antipyretics included: paracetamol and combinations (Panadol, Curamol, Painex, Dynapar, Ibupar, Action, Kamadol, Metopar), ibuprofen, diclofenac, piroxicam, tramadol, aspirin, indomethacin, meloxicam, and amitriptyline.

Blood samples were collected from 253 index clients with an unknown malaria status at the drug shop, out of 351 eligible clients (72%). There were significant associations between consenting to blood sample collection and sex [$X_{\left(1\right)}^{2}$ = 6.09, *p* = 0.014], as well as study group [$X_{\left(3\right)}^{2}$ = 127.6, *p* < 0.001]. Women and clients from later study groups were more likely to provide a blood sample than men or clients from study group one. There were no significant differences by age or days of illness. Among 221 clients treated presumptively for malaria (i.e., unknown status at the drug shop and purchased an antimalarial), 65% tested negative based on RDTs conducted on collected blood samples (Figure 1). Nearly all blood samples from clients with an unknown status who did not purchase an antimalarial (*n* = 32) tested negative (*n* = 31, 97%).

### 3.3. Predictors of rapid diagnostic test uptake

Client demographic characteristics, vendor qualifications, RDT cost, and time of visit were not significant predictors of having a known malaria status at the drug shop (Table 4). Days of illness, drug shop location, and shop years of operation predicted RDT use in univariate models, but significance was not maintained in multivariate models. Study group and day of visit were associated with RDT use in both univariate and multivariate models. Clients who came to the drug shop on a weekday were 1.25 times as likely (95% CI: 1.02, 1.54) to know their malaria test status than clients who came over the weekend. Compared to study group 3 (Katooke trading center), clients in study group 1 (Bugoye trading center) were 1.88 times as likely (95% CI: 1.40, 2.52), and clients in study group 2 (Ibanda trading center) were 2.43 times as likely (95% CI: 1.82, 3.24) to know their malaria status at the drug shop.

**Table 4:** Estimated risk ratios from univariate and multivariate log binomial regression modeling of having a known malaria RDT status^*^ among clients seeking malaria treatment from drug shops in Bugoye sub-county, Uganda^†^.

	**Univariate regression**			**Multivariate regression**		
	**RR**	**95% CI**	***p*** **-value**	**aRR**	**95% CI**	***p*** **-value**
**Study group**						
Group 1—Bugoye	1.93	1.46, 2.55	<0.0001	1.88	1.40, 2.52	<0.0001
Group 2—Ibanda	2.59	1.98, 3.38	<0.0001	2.43	1.82, 3.24	<0.0001
Group 3—Katooke	Ref.			Ref.		
Group 4—Kisamba	1.19	0.83, 1.70	0.3398	1.15	0.76, 1.74	0.5024
**Sex of client**						
Male	Ref.			Ref.		
Female	1.09	0.93, 1.27	0.3085	1.06	0.91, 1.25	0.4448
**Age of client** ^‡^						
5 years	0.97	0.79, 1.19	0.7794	1.29	0.95, 1.72	0.1027
15 years	0.98	0.84, 1.14	0.7794	1.24	0.95, 1.61	0.1096
30 years	0.99	0.92, 1.07	0.7794	1.17	0.94, 1.46	0.1517
45 years	Ref.			Ref.		
**Days of illness** ^‡^						
1 day	Ref.			Ref.		
3 days	1.06	1.04, 1.08	<0.0001	1.15	0.94, 1.42	0.1820
7 days	1.19	1.12, 1.27	<0.0001	1.23	0.99, 1.54	0.0672
**Drug shop location** ^§^						
Small trading center	Ref.			Ref.		
Large trading center	1.43	1.22, 1.67	<0.0001	1.08	0.88, 1.34	0.4585
**Drug shop years of operation** ^‡^						
1 year	Ref.			Ref.		
5 years	1.09	1.02, 1.16	0.0076	1.16	0.93, 1.44	0.1889
10 years	1.21	1.05, 1.39	0.0076	1.21	0.93, 1.58	0.1531
**Vendor training**						
Nursing assistant	Ref.			Ref.		
Nurse or midwife	1.04	0.88, 1.24	0.6381	1.04	0.86, 1.25	0.6919
**Malaria RDT cost** ^‡^						
2,000 UG^#^	0.93	0.80, 1.08	0.3167	0.99	0.76, 1.30	0.9662
3,000 UGX^¶^	Ref.			Ref.		
**Day of drug shop visit**						
Weekday	1.26	1.03, 1.54	0.0269	1.25	1.02, 1.54	0.0339
Weekend	Ref.			Ref.		
**Time of drug shop visit**						
Morning	1.06	0.86, 1.31	0.5809	0.98	0.78, 1.21	0.8233
Afternoon	1.22	1.00, 1.48	0.0512	1.12	0.91, 1.37	0.2971
Evening	Ref.			Ref.		

* Includes both RDTs conducted prior to drug shop visit and RDTs conducted at the drug shop.

† From 934 clients, data from 840 with no missing values were used in the adjusted analysis.

‡ Age of client, days of illness, drug shop years of operation, and malaria RDT cost were modeled as continuous variables. RRs were calculated from select, illustrative values.

§ Large trading centers were defined by the presence of a weekly market day.

# Approximately 0.57 USD.

¶ Approximately 0.85 USD.

## 4. Discussion

While the Uganda Ministry of Health has adopted the WHO guidelines of “test, treat, and track” for all suspected malaria cases, this study demonstrates that despite widespread RDT availability, individuals purchasing antimalarials from drug shops were often not tested to confirm a malaria diagnosis (60%). When RDTs were conducted, clients who tested negative sometimes still purchased antimalarials (36%). Qualitative research in this setting found that these antimalarial purchases stemmed from vendor and client distrust in negative RDT results, and fear or uncertainty about treatment next steps for conditions other than malaria (41).

The low RDT use at drug shops in our study is especially concerning because vendors and clients could not distinguish between malaria and other causes of fever, and most clients treated presumptively did not have malaria (65%). This confirms malaria overtreatment at drug shops in Bugoye sub-county is substantial, with potential consequences for individual health, economic status, and population-level parasite resistance.

While previous interventions in Uganda reduced malaria overtreatment by introducing RDTs into drug shops (42–45), these tests are readily available in Bugoye sub-county. These programs also relied on the provision of free or subsidized RDTs. However, our study results suggest that this strategy alone would be insufficient to improve practices. It was common in Bugoye for surrogate clients to seek treatment for another person-−30% of index clients were not present at the drug shop, a finding aligned with previous studies in Uganda (30, 46). If the sick individual is at home, increased availability and lower cost of RDTs at drug shops will not increase their use. Furthermore, antimalarial purchases after testing negative suggest interventions that promote trust and understanding of RDT results are needed.

Our analysis did not identify many modifiable predictors of RDT use. Clients visiting drug shops on weekends, when public health facilities are closed, were less likely to receive an RDT. This suggests longer and more flexible operating hours at public facilities could increase the proportion of drug shop clients with a known malaria status. Differences by study group may be explained by the timing and location of data collection. Study groups were chosen based on geographic proximity, and access to health centers and socio-economic status (not measured in this study) vary across the sub-county.

This study also raises concerns about the types of medications sold at drug shops. While the most common antimalarial purchased by drug shop clients in our study was artemether/lumefantrine (53%), the first-line ACT in Uganda (47), clients also purchased antimalarials no longer recommended in Uganda due to high levels of resistance, such as sulfadoxine/pyrimethamine (13%) and quinine (8%) (48–50). Clients also purchased both antimalarials and antibiotics (28%), regardless of RDT purchases and results. Antibiotic misuse fuels resistance, with global implications for the effective treatment of infectious diseases (51).

Poor malaria case management at drug shops in Bugoye sub-county may be related to inadequate training and experience, which can be reflected in low vendor knowledge (52, 53). Most drug shops in Bugoye were unlicensed and operated by vendors without the necessary qualifications to operate a drug shop in Uganda, a trend consistent across sub-Saharan Africa (54). Despite concerns, drug shops are a reliable source of essential medications. While services and medications from the public sector are free, drug shops are appealing because they have convenient locations and hours, short wait times, and infrequent drug stockouts (24, 55). Given the important role drug shops play in community health, and the continued persistence of malaria as a leading cause of morbidity and mortality, interventions are needed to improve malaria case management in the Ugandan private sector.

### 4.1. Limitations

This study may overestimate RDT use. Evaluating drug shop practices without influencing vendor or client behavior is a recognized challenge for studies with private medicine retailers, because behavior may change when individuals are aware they are being monitored (42, 56). Vendor data collection was chosen over direct observation or exit interviews to minimize research participation effects. However, vendors may have been more likely to stock or promote RDTs, or insist on adherence to results because of study participation. Additionally, prior RDT results were self-reported by clients and subject to social-desirability bias. This study also relied on vendors to accurately record information. Data collection in groups allowed for more control over data quality and forms were reviewed triweekly. No systemic issues in reporting were detected and completeness was high, with missing values <5%. Finally, blood samples were only obtained if the index client was present and provided consent, and the data collection period was short. Therefore, data may not provide a complete representation of malaria diagnostic and treatment practices at drug shops in Bugoye. Nevertheless, estimates provide a useful starting point to understanding the magnitude of the problem, a prerequisite to proposing solutions.

## 5. Conclusion

This study is the first to quantify RDT use and malaria overtreatment at drug shops in Bugoye sub-county. Since drug shops provide a substantial percentage of antimalarials (>50%), future interventions to improve malaria case management at drug shops could increase rational drug use, reduce unnecessary spending, and delay the development of parasite resistance to ACTs. The consequences of malaria misdiagnosis disproportionately affect the poor and vulnerable, contributing to a cycle of disease and poverty (18). Therefore, efforts to improve fever case management and diagnostic practices at private drug shops could have tangible social and economic benefits. Since challenges related to malaria overtreatment and the quality of care at drug shops are not unique to Bugoye, findings could be relevant for other low- and middle-income countries.

## Data availability statement

The raw data supporting the conclusions of this article will be made available by the authors, without undue reservation.

## Ethics statement

The studies involving human participants were reviewed and approved by the University of North Carolina Office of Human Research Ethics (20-3019), Mbarara University of Science and Technology Research Ethics Committee (MUST-2021-55), and the Uganda National Council for Science and Technology. The patients/participants or participants' legal guardian/next of kin provided their written informed consent to participate in this study.

## Author contributions

VS, EM, CB, and RB contributed to conception and design of the study. NM was responsible for all project administration. VS, NM, and EB trained drug shop vendors. VS conducted the analysis and wrote the original draft of the manuscript. All authors contributed to manuscript revisions and approved the submitted version.
