# Supplementary material for: Private sector antimalarial sales a decade after “test and treat”: A cross-sectional study of drug shop clients in rural Uganda
Source: Front Public Health. 2023 Mar 28;11:1140405. doi: 10.3389/fpubh.2023.1140405 (PMC10089286; doi:10.3389/fpubh.2023.1140405)
Supplement: Supplementary file 1 [file Data_Sheet_1.docx]

Supplementary Material

Private sector antimalarial sales a decade after “test and treat”: A cross-sectional study of drug shop clients in rural Uganda

**Victoria Shelus, Nobert Mumbere, Edgar M. Mulogo, Clare Barrington, Emmanuel Baguma, Rabbison Muhindo, James E. Herrington Jr., Michael Emch, Suzanne Maman, Ross M. Boyce**

*** Correspondence:** Ross M. Boyce: ross_boyce@med.unc.edu

# Initial drug shop survey

| **1. Drug Shop Information** | |
| --- | --- |
| Date of visit |  |
| Village |  |
| Drug Shop Name |  |
| GPS Coordinates |  |
| Elevation |  |
| Picture |  |
| Description of location *(for study team identification)* |  |

**Study description:** We are conducting a study to understand malaria diagnosis and treatment provided by drug shops in Bugoye. This is a follow-up to previous research in 2017 that you may have participated in. We are reaching out to all drug shops in Bugoye to see if they would be willing to participate. Data collection will take place for two weeks. During that time, we would like you to collect basic information from all clients complaining of fever or requesting antimalarials. If the individual with fever is present at the drug shop, we would like you to collect a blood sample into a collection tube. These procedures are estimated to take less than 5 minutes per client, and we will train you and provide all the materials that you need. You will receive a small stipend for participating in this study.

| **2. Study Participation: Interest and Eligibility** | | |
| --- | --- | --- |
| Are you interested in learning more about participating in this study? | 0 | No 🡪 END |
|  | 1 | Yes |
| Are you willing to participate in a short training about study procedures? | 0 | No 🡪 END |
|  | 1 | Yes |

| **3. Malaria RDTs** | | | |
| --- | --- | --- | --- |
| Do you sell malaria RDTs in your shop? | 0 | No 🡪 Skip to next section | |
|  | 1 | Yes | |
| How much do you charge for a malaria RDT? |  | | |
| How many boxes of RDTs do you currently have? |  | | |
| Box 1 | Type | |  |
|  | Number remaining | |  |
|  | Expiration date | |  |
| Box 2 | Type | |  |
|  | Number remaining | |  |
|  | Expiration date | |  |
| Box 3 | Type | |  |
|  | Number remaining | |  |
|  | Expiration date | |  |
| Box 4 | Type | |  |
|  | Number remaining | |  |
|  | Expiration date | |  |

| **4. Additional Drug Shop Information** | | |
| --- | --- | --- |
| Approximately how many clients visited your shop last week? |  | |
| Approximately how many of those clients visited your shop for fever or malaria treatment? |  | |
| How long has this drug shop been operating? |  | |
| Does the drug shop have a current registration/license? | 0 | No |
|  | 1 | Yes |
|  | 2 | Prefer not to say |
|  | 3 | Unsure |
| Are you the owner of the drug shop? | 0 | No |
|  | 1 | Yes 🡪 Skip next question |
| What is the background of the owner of the drug shop? | 1 | Pharmacy technician/dispenser* |
|  | 2 | Nurse (registered or enrolled)* |
|  | 3 | Comprehensive nurse* |
|  | 4 | Midwife (registered or enrolled)* |
|  | 5 | Nursing assistant |
|  | 6 | Medical doctor |
|  | 7 | Other health professional, specify: _______________ |
|  | 8 | Other non-medical professional, specify: _______________ |
| What is your background? | 1 | Pharmacy technician/dispenser* |
|  | 2 | Nurse (registered or enrolled)* |
|  | 3 | Comprehensive nurse* |
|  | 4 | Midwife (registered or enrolled)* |
|  | 5 | Nursing assistant |
|  | 6 | Medical doctor |
|  | 7 | Other health professional, specify: _______________ |
|  | 8 | Other non-medical professional, specify: _______________ |

| **5. Contact Information** | | |
| --- | --- | --- |
| How many people (including you) work in this shop? |  | |
| Owner | Name |  |
|  | Phone number |  |
| Worker 1 | Name |  |
|  | Phone number |  |
| Worker 2 | Name |  |
|  | Phone number |  |
| Worker 3 | Name |  |
|  | Phone number |  |
| Worker 4 | Name |  |
|  | Phone number |  |

# Data collection form

Drug Shop Name: ______________________________________________________________

Drug Shop ID: ________ -- ________ -- ________ Client ID:________ -- ________ -- ________

Date (day/month):______________________ Time: ______________________

| *CLIENT DEMOGRAPHICS* | | | | | | | |
| --- | --- | --- | --- | --- | --- | --- | --- |
| **1. Is the person who is sick at the drug shop?** | | | | | | | |
| 🞎Yes | | | 🞎No  Who is at the drug shop? | | | | |
|  |  |  | 🞎Mother | 🞎Father | 🞎Wife | | 🞎Husband |
|  |  |  | 🞎Daughter | 🞎Son | 🞎Sister | | 🞎Brother |
|  |  |  | 🞎Friend/Neighbor | | 🞎Other: __________________ | | |
| **2. Client’s village:** | | | | | | | |
| **3. Client’s age** *(Estimate if exact age unknown):* | | | | | | | |
| **4. Client’s sex** | | | | | | | |
| 🞎Male | | | 🞎Female | | | | |
|  |  |  | Is the client pregnant? | |  | | |
|  |  |  | 🞎No | | 🞎Yes, # months ____________ | | |
| *ILLNESS HISTORY* | | | | | | | |
| **5. Number of days client has been sick:** | | | | | | | |
| **6. Symptoms** (*Do NOT read options out loud. Select all that are mentioned.)* | | | | | | | |
| 🞎Fever | 🞎Fatigue | | 🞎Joint pain | 🞎Muscle pain | 🞎Headache | | 🞎Shivering |
| 🞎Nausea | 🞎Diarrhoea | | 🞎Cough | 🞎Sore throat | 🞎Loss of taste/smell | | |
| 🞎Other(s):_________________________________________________________________________ | | | | | | | |
| *TEST RESULTS* | | | | | | | |
| **7. Has the client had a malaria RDT within the last few days?** | | | | | | | |
| 🞎No | | | 🞎Yes, positive RDT | | 🞎Yes, negative RDT | | |
|  |  |  | Where was the RDT conducted? | | | | |
|  |  |  | 🞎Bugoye | 🞎RMS | 🞎Nyangonge | | 🞎Kibirizi |
|  |  |  | 🞎Kisamba | 🞎Maghoma | 🞎Katooke | | 🞎Ibanda |
|  |  |  | 🞎Bughaghura | 🞎VHT | 🞎Other: _______________ | | |
| **8. Was an RDT conducted at the drug shop today?** | | | | | | | |
| 🞎No | | 🞎Yes, positive result | | 🞎Yes, negative result | | 🞎Client took test to conduct somewhere else. | |
| *MEDICATIONS* | | | | | | | |
| **9. Drugs purchased** *(Select all)* | | | | | | | |
| 🞎None | 🞎Coartem | | 🞎Lonart | 🞎Fansidar | 🞎P-Alaxin | | 🞎Duo-Cotecxin |
| 🞎Quinine tabs | 🞎Quinine IV | | 🞎Quinine syrup | 🞎Artesunate | 🞎Camoquin | | 🞎Chloroquine |
| 🞎Panadol | 🞎Ibuprofen | | 🞎Diclofenac | 🞎Piroxicam | 🞎Aspirin | | 🞎Painex |
| 🞎Curamol | 🞎Dynapar | | 🞎Tramadol | 🞎Amoxicillin | 🞎Ampicillin | | 🞎Erythromycin |
| 🞎Other(s):_________________________________________________________________________ | | | | | | | |
| *BLOOD SAMPLE* | | | | | | | |
| **10. Was a blood sample collected?** | | | | | | | |
| 🞎Yes | | 🞎No | | | | | |
|  |  | Why was a blood sample not collected? | | | | | |
|  |  | 🞎Client did not consent | | 🞎Person who is sick is not at the drug shop | | 🞎RDT conducted at drug shop | |

# Specific medication purchases of drug shop clients

|  |  | All clients  N=934 | |  | Tested positive for malaria*  N=202 | |  | Tested negative for malaria*  N=171 | |  | Did not test  N=561 | |
| --- | --- | --- | --- | --- | --- | --- | --- | --- | --- | --- | --- | --- |
|  |  | n | % |  | n | % |  | n | % |  | n | % |
| Any antimalarial |  | 741 | 79.3 |  | 189 | 93.6 |  | 62 | 36.3 |  | 490 | 87.3 |
| Artemether/  lumefantrine |  | 499 | 53.4 |  | 120 | 59.4 |  | 24 | 14.0 |  | 355 | 63.3 |
| Sulfadoxine/  pyrimethamine |  | 118 | 12.6 |  | 7 | 3.5 |  | 29 | 17.0 |  | 82 | 14.6 |
| Artesunate |  | 34 | 3.6 |  | 24 | 11.9 |  | 3 | 1.8 |  | 7 | 1.2 |
| Quinine (IV) |  | 26 | 2.8 |  | 14 | 6.9 |  | 1 | 0.6 |  | 11 | 2.0 |
| Quinine (tabs) |  | 25 | 2.7 |  | 12 | 5.9 |  | 1 | 0.6 |  | 12 | 2.1 |
| Quinine (syrup) |  | 25 | 2.7 |  | 7 | 5.9 |  | 1 | 0.6 |  | 17 | 3.0 |
| Dihydroartemisinin  /piperaquine |  | 19 | 2.0 |  | 7 | 3.5 |  | 3 | 1.8 |  | 9 | 1.6 |
| Chloroquine |  | 1 | 0.1 |  | 1 | 0.5 |  | 0 | 0.0 |  | 0 | 0.0 |
|  |  |  |  |  |  |  |  |  |  |  |  |  |
| Any antibiotic |  | 386 | 41.3 |  | 85 | 42.1 |  | 119 | 69.6 |  | 182 | 32.4 |
| Amoxicillin |  | 240 | 25.7 |  | 56 | 27.7 |  | 66 | 38.6 |  | 118 | 21.0 |
| Ampicillin |  | 58 | 6.2 |  | 11 | 5.4 |  | 18 | 10.5 |  | 29 | 5.2 |
| Erythromycin |  | 51 | 5.5 |  | 8 | 4.0 |  | 19 | 11.1 |  | 24 | 4.3 |
| Metronidazole |  | 26 | 2.8 |  | 6 | 3.0 |  | 6 | 3.5 |  | 14 | 2.5 |
| Co-trimoxazole |  | 15 | 1.6 |  | 2 | 1.0 |  | 5 | 2.9 |  | 8 | 1.4 |
| Ciprofloxacin |  | 12 | 1.3 |  | 4 | 2.0 |  | 6 | 3.5 |  | 2 | 0.4 |
| Ceftriaxone |  | 10 | 1.1 |  | 1 | 0.5 |  | 7 | 4.1 |  | 2 | 0.4 |
| Gentamicin |  | 4 | 0.4 |  | 0 | 0.0 |  | 3 | 1.8 |  | 1 | 0.2 |
| Penicillin V |  | 3 | 0.3 |  | 0 | 0.0 |  | 2 | 1.2 |  | 1 | 0.2 |
| Amplicox |  | 3 | 0.3 |  | 1 | 0.5 |  | 1 | 0.6 |  | 1 | 0.2 |
| Benzylpenicillin |  | 2 | 0.2 |  | 0 | 0.0 |  | 2 | 1.2 |  | 0 | 0.0 |
| Cefalexin |  | 2 | 0.2 |  | 0 | 0.0 |  | 2 | 1.2 |  | 0 | 0.0 |
| Tinidazole |  | 2 | 0.2 |  | 1 | 0.5 |  | 0 | 0.0 |  | 1 | 0.2 |
| Azithromycin |  | 1 | 0.1 |  | 0 | 0.0 |  | 1 | 0.6 |  | 0 | 0.0 |
| Chloramphenical |  | 1 | 0.1 |  | 1 | 0.5 |  | 0 | 0.0 |  | 0 | 0.0 |
|  |  |  |  |  |  |  |  |  |  |  |  |  |
| Any analgesic /antipyretic |  | 830 | 88.9 |  | 167 | 82.7 |  | 158 | 92.4 |  | 505 | 90.0 |
| Paracetamol^†^ |  | 588 | 63.0 |  | 125 | 61.9 |  | 96 | 56.1 |  | 367 | 65.4 |
| Ibuprofen |  | 108 | 11.6 |  | 24 | 11.9 |  | 26 | 15.2 |  | 58 | 10.3 |
| Diclofenac |  | 102 | 10.9 |  | 15 | 7.4 |  | 24 | 14.0 |  | 63 | 11.2 |
| Piroxicam |  | 45 | 4.8 |  | 12 | 5.9 |  | 15 | 8.8 |  | 18 | 3.2 |
| Tramadol |  | 7 | 0.7 |  | 0 | 0.0 |  | 2 | 1.2 |  | 5 | 0.9 |
| Aspirin |  | 3 | 0.3 |  | 0 | 0.0 |  | 1 | 0.6 |  | 2 | 0.4 |
| Indomethacin |  | 1 | 0.1 |  | 0 | 0.0 |  | 0 | 0.0 |  | 1 | 0.2 |
| Meloxicam |  | 1 | 0.1 |  | 0 | 0.0 |  | 1 | 0.6 |  | 0 | 0.0 |
| Amitriptyline |  | 1 | 0.1 |  | 0 | 0.0 |  | 1 | 0.6 |  | 0 | 0.0 |

*Includes both RDTs conducted prior to drug shop visit and RDTs conducted at the drug shop.

†Paracetamol and combinations (Panadol, Curamol, Painex, Dynapar, Ibupar, Action, Kamadol, Metopar)
